# The predictive value of risk assessment models for venous thromboembolism on gynaecological cancer patients

**DOI:** 10.4102/jcmsa.v3i1.164

**Published:** 2025-08-05

**Authors:** Phuti Khomotso Ratshabedi, Admire Chikandiwa, Langanani Mbodi

**Affiliations:** 1 Department of Obstetrics and Gynaecology, Faculty of Clinical Medicine, University of the Witwatersrand, Johannesburg, South Africa

**Keywords:** risk assessment models, Caprini score, Padua score, Khorana score, venous thromboembolism, gynaecological cancers

## Abstract

**Background:**

Venous thromboembolism (VTE) is a multifactorial disease. There are two main clinical entities that are associated with morbidity and mortality, deep vein thrombosis and pulmonary embolism. Our study aimed to compare the three risk assessment models (RAMs), Khorana, Caprini and Padua in terms of predicting VTE in gynaecologic oncology patients.

**Methods:**

Patients were retrospectively scored according to Caprini, Padua and Khorana scoring models to assess the risk for VTE. Accuracy analysis of risk assessment models was performed using sensitivity, specificity, positive and negative predictive values as well as the area under the curve of each model per patient.

**Results:**

The Caprini score has good sensitivity (80.0), a poor specificity (24.3), low positive predictive value (7.2) and good negative predictive value (94.3) (95% CI). The Khorana score has a poor sensitivity (30.0), a fair specificity (62.5), low positive predictive value (5.6) and good negative predictive value (92.4) (95% CI). The Padua score has an average sensitivity (60.0), a poor specificity (42.6), low positive predictive value (7.1) and good negative predictive value (93.5) (95% CI). The Caprini score had the overall best performance.

**Conclusion:**

Caprini score performed better and proved to be the best score. It has the potential to reduce mortality associated with VTE in gynaecological cancer patients. However, the Caprini score needs to be tested in the same population in a prospective study in a multicentre.

**Contribution:**

The results of this study prove to us that Caprini score is the best to be used in a South African setting.

## Introduction

Venous thromboembolism (VTE) is a multifactorial disease. Two main clinical entities are associated with morbidity and mortality, for example, deep vein thrombosis (DVT) and pulmonary embolism (PE).^1^ Venous thromboembolism carries a significant burden and has been observed as a common and frequent complication in patients with active cancer. Its overall 12-month incidence is estimated to be approximately 6% – 8% although it varies widely across cancer types.^2^ Venous thromboembolism is associated with significant morbidity and mortality and reduces the quality of life in patients. Both PE and DVT are commonly detected with the aid of CT scan and ultrasound scan preoperatively in cancer patients with 11.8% DVT and 4.7% PE. In asymptomatic patients, screening with radiology (computerised tomography and ultrasound) revealed as much as 86.7% DVT and 30.8% PE. These figures show how common these diseases can be in cancer patients.^3^

### Literature review

The incidence of VTE is spread equally across the demographics with no significant difference in terms of mean age, mean body mass index (BMI), comorbidities (i.e. hypertension and diabetes), mean tumour diameter, histological type of cancer, or operative time for those who are postoperative. However, the incidence of DVT is significantly higher in patients who developed previous cancer (*p* = 0.047), who were in advanced stage (*p* = 0.001), received radiotherapy (*p* < 0.001), received chemotherapy (*p* < 0.001), underwent lymphadenectomy (*p* = 0.014) and those who had a massive operative blood loss ≥1500 mL (*p* = 0.003). The median time for occurrence of DVT after diagnosis of cancer is 4 months (interquartile range [IQR]: 2–12) with the majority (78.2%) occurring within the first 12 months of cancer diagnosis. The incidence also peaks at periods 1–2 years after diagnosis of cancer.^4^

The Khorana Score is the most used risk assessment model (RAM) designed to stratify cancer outpatients before the start of chemotherapy. It is believed to be a simple and user-friendly tool that combines routinely available parameters to assign patients to different classes of VTE risk. The Khorana score of 3 was initially proposed in a thromboprophylaxis guidance statement but later studies showed its low sensitivity for certain tumour types.^1^ To improve its predictive performance, the original Khorana score was modified by adding either chemotherapy agents, such as platinum-based regimens and gemcitabine, as in the case of the PROTECT score, which resulted in an improved ability to identify patients at higher risk for VTE, or biomarkers.^1^

In the meta-analysis, Mulder et al. found that the incidence of VTE in the first 6-month period was 5.0% (95% CI: 3.9–6.5) in patients with a low-risk Khorana score (0 points), 6.6% (95% CI: 5.6–7.7) in those with an intermediate-risk Khorana score (1 or 2 points) and 11.0% (95% CI: 8.8–13.8) in those with a high-risk Khorana score (3 points or higher). (7) The summary incidence of VTE was 5.7% (95% CI: 4.2–7.9) in patients with a low-risk Khorana score (0 points), 8.6% (95% CI: 7.3–10.2) in those with an intermediate-risk Khorana score (1 or 2 points) and 14.0% (95% CI:11.7–16.7) in those with a high-risk Khorana score (3 points or higher).^2^ The Khorana score appears to be less informative for lung and haematological malignancies. This score is of limited use in ruling out a future venous thromboembolic event. Lastly, the Khorana score is designed to select patients in the high-risk group for thromboprophylaxis.^2^

The risk scores of VTE patients were significantly higher than those of non-VTE patients based on both the Caprini and Padua RAMs (both *p* < 0.01). The Caprini RAM found that the majority of the VTE patients (70.9%) were classified to be high–superhigh risk level, whereas most of non-VTE patients (73.4%) were classified as low–moderate risk level (*p* = 0.001). The Padua RAM found only 23.4% of VTE patients and 14.4% of non-VTE patients to be at high risk for VTE (*p* = 0.003). The sensitivity and positive and negative predictive values (NPVs) of the Caprini RAM were also higher than that of the Padua RAM (all *p* < 0.05) although the specificity was lower than that of the Padua RAM (*p* = 0.001).^5^ These calculations were based on hospitalised medical patients. The study concluded that the implementation of Caprini RAM may improve the efficiency of prophylaxis and decrease the incidence of VTE in the clinical setting.^5^

The VTE risk increased significantly with an increase in the cumulative Padua Prediction Score (PPS) or Caprini RAM score. A PPS and Caprini RAM ‘high risk’ classification was associated with a 5.01-fold and 4.10-fold increased VTE risk, respectively. The Caprini RAM could identify 84.3% of the VTE cases to receive prophylaxis according to American College of Chest Physicians guidelines, and the PPS could only identify 49.1% of the VTE cases.^6^

Both models can be used to stratify the VTE risk in medical inpatients effectively, but the Caprini RAM may be considered as the first choice in a general hospital because it incorporates comprehensive risk factors, higher sensitivity and potential for prediction of mortality.^6^

The study aimed to compare the three RAMs, Khorana, Caprini and Padua in terms of predicting the VTE in gynaecologic oncology patients.

## Methods

**FIGURE 1:**
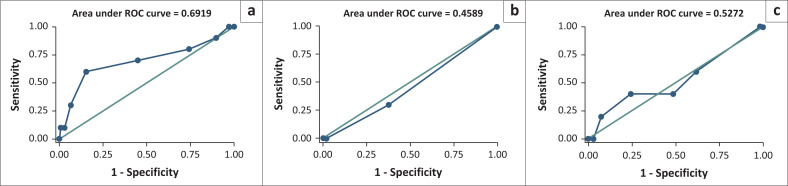
Receiver operating characteristics curves: (a) Caprini (b) Khorana (c) Padua.

Files from gynaecological department for hospitalised patients were retrieved dating from 01 January 2017 to 31 December 2019. Patients were randomly sampled. Descriptive analyses were conducted using frequencies and percentages for categorical variables. Continuous variables were analysed by measures of central tendency (means with standard deviations and medians with IQR). Patients were then scored in retrospect according to Caprini, Padua and Khorana scoring models to assess the risk for VTE. Accuracy analysis of RAMs was performed using sensitivity, specificity, positive (PPV) and negative predictive value (NPV) of each model per patient. For the purposes of this analysis, the following cut-off scores were used: ≥ 5 for the Caprini RAM, ≥ 4 for the Padua score and ≥ 2 for the Khorana RAM. Receiver operator characteristics (ROC) curves were also plotted (see Figure 1) and area under the curve was used to determine the overall discriminating power of a screening test. Factors associated with VTE were explored using logistic regression models. Data analyses were conducted in Stata 15.0® (StataCorp, 4905 Lakeway Drive, College Station, Texas 77845 USA).

### Ethical considerations

Ethical clearance to conduct this study was obtained from the University of the Witwatersrand Human Research Ethics Committee (WHREC) (M123450) and was registered with the National Health Research Database (Reference: 123450).

## Results

The study retrospectively recruited 146 gynaecologic oncology patients who were admitted over the period of 2 years in an academic centre in Johannesburg, South Africa. Of these, 10 were confirmed to have VTE diseases while 1 patient was treated for VTE but then later excluded on radiology. The VTE risk scoring tools were retrospectively applied to assess if they would have predicted the disease and their sensitivity, specificity, NPV, and PPV were calculated. Other variables were also described.

### Caprini score

For the Caprini RAM, a cut-off score of greater than or equal to 5 had a sensitivity of 80%, a specificity of 24%, a PPV of 7% and a NPV of 94% (Table 1).

**TABLE 1:** Performance of Caprini score in predicting venous thromboembolism.

Diagnosis	Caprini score		Total	Sensitivity		Specificity		Positive predictive value		Negative predictive value		AUC	
	≥ 5	< 5		Score	95% CI	Score	95% CI	Score	95% CI	Score	95% CI	Score	95% CI
**VTE diagnosis**	-	-	-	80.0	44.4–97.5	24.3	17.3–32.4	7.2	3.2–13.7	94.3	80.8–99.3	0.52	0.39–0.66
Positive	8	2	10	-	-	-	-	-	-	-	-	-	-
Negative	103	33	136	-	-	-	-	-	-	-	-	-	-

**Total**	**111**	**35**	**146**	**-**	**-**	**-**	**-**	**-**	**-**	**-**	**-**	**-**	**-**

VTE, Venous thromboembolism; AUC, area under the curve.

### Khorana score

For the Khorana RAM, a cut-off score of greater than or equal to 2 had a sensitivity of 30%, a specificity of 62%, a PPV of 5% and a NPV of 92% (Table 2).

**TABLE 2:** Performance of Khorana Score in predicting venous thromboembolism.

Diagnosis	Khorana score		Total	Sensitivity		Specificity		Positive predictive value		Negative predictive value		AUC	
	≥ 2	< 2		Score	95% CI	Score	95% CI	Score	95% CI	Score	95% CI	Score	95% CI
**VTE diagnosis**	-	-	-	30.0	6.7–65.2	62.5	53.8–70.6	5.6	1.2–15.4	92.4	84.9–96.9	0.46	0.31–0.62
Positive	3	7	10	-	-	-	-	-	-	-	-	-	-
Negative	51	85	136	-	-	-	-	-	-	-	-	-	-

**Total**	**54**	**92**	**146**	**-**	**-**	**-**	**-**	**-**	**-**	**-**	**-**	**-**	**-**

VTE, Venous thromboembolism; AUC, area under the curve.

### Padua score

For the Padua RAM, a cut-off score of greater than or equal to 4 had a sensitivity of 60%, a specificity of 42%, a PPV of 7% and a NPV of 93% (Table 3).

**TABLE 3:** Performance of Padua Score in predicting venous thromboembolism.

Diagnosis	Padua score		Total	Sensitivity		Specificity		Positive predictive value		Negative predictive value		AUC	
	≥ 4	< 4		Score	95% CI	Score	95% CI	Score	95% CI	Score	95% CI	Score	95% CI
**VTE diagnosis**	-	-	-	60.0	26.2–87.8	42.6	34.2–51.4	7.1	2.7–14.9	93.5	84.3–98.2	0.51	0.35–0.51
Positive	6	4	10	-	-	-	-	-	-	-	-	-	-
Negative	78	58	136	-	-	-	-	-	-	-	-	-	-

**Total**	**84**	**62**	**146**	**-**	**-**	**-**	**-**	**-**	**-**	**-**	**-**	**-**	**-**

VTE, venous thromboembolism; AUC, area under the curve.

The ROC curve analysis shows that a score of 7 and above gives the best performance with 60% sensitivity and 85% specificity for Caprini RAM while a Padua RAM score of 4 or more gives the best balance between sensitivity (60%) and specificity (38%). Overall, the ROC analysis showed that Khorana RAM was a poor predictor of VTE with AUC less than 0.5.

### Demographic, clinical factors and risks for venous thromboembolism (above the cancer diagnosis)

Most of the patients in our cohort were of African ethnicity and postmenopausal. Few patients did not have a confirmed comorbid condition and those who had, were well controlled (Table 4 and Table 5).

**TABLE 4:** Baseline demographic factors (*N* = 146).

Description	*n*	%	Mean	s.d.
Age†	52.6	13.9	-	-
**Ethnicity**				
Black people	103	89.6	-	-
White people	1	0.9	-	-
Coloured people	11	9.6	-	-
Parity‡	-	-	2.4	1.7
**Parity category**				
0–1	42	28.9	-	-
2+	103	71.0	-	-
**Menopausal status**				
Pre	42	28.9	-	-
Post	103	71.0	-	-
Unknown	53	36.3	-	-

IQR, interquartile range.

† , Median = 51.5; IQR, range = 42–64, 19–91;

‡ , Median = 2; IQR, range = 1–3, 0–9.

**TABLE 5:** Baseline risk factors and comorbid diseases (*N* = 146).

Description	*n*	%	Mean	s.d.
**HPT (*n* = 145)**				
Yes	61	42.1	-	-
No	84	57.9	-	-
**HPT control status (*n* = 61)**				
Yes	48	78.7	-	-
No	13	21.3	-	-
**DM (*n* = 145)**				
Yes	18	12.4	-	-
No	127	87.6	-	-
**DM control status (*n* = 18)**				
Yes	15	83.3	-	-
No	3	16.7	-	-
**Cardiac disease (*n* = 146)**				
Yes	6	4.1	-	-
No	140	95.9	-	-
**Cardiac disease control status (*n* = 6)**				
Yes	2	33.3	-	-
No	6	66.7	-	-
**Hypercholesterolaemia (*n* = 145)**				
Yes	11	7.6	-	-
No	134	92.4	-	-
**Hypercholesterolaemia control status (*n* = 11)**				
Yes	11	100.0	-	-
No	0	0.0	-	-
**HIV**				
Positive	-	-	47.0	31.2
Negative	-	-	99.0	67.8
CD4+ count (*n* = 42)†	-	-	417.1	249.0
HIV VL undetectable (*n* = 40)	-	-	31.0	77.5

IQR, interquartile range; VL, viral load.

† , Median = 401; IQR, range = 222–510, 42–1200.

The majority of confirmed VTEs were DVTs diagnosed through the Doppler scan on patients who presented with a swollen leg (Table 6).

**TABLE 6:** Venous thromboembolism treatment (*N* = 11) and venous thromboembolism diagnosis (*N* = 10).

Description	*n*	%
**Anti-coagulation before diagnosis**		
Yes	7	63.6
No	4	36.4
**Type of drug (*n* = 7)**		
LMWH	7	100.0
**Type of dosing (*n* = 7)**		
Therapeutic	7	0.0
Duration of pre-diagnosis treatment (weeks)†	1	0.0
**VTE type (*n* = 10)**		
DVT	7	70.0
PE	2	20.0
Prehepatic VTE (incidental on staging CT)	1	10.0
**Method of diagnosing VTE**		
Doppler Scan	6	54.5
CT Scan	4	36.4
CTPA	1	9.1
**Reasons for investigating the VTE**		
Swollen leg	8	-
SOB	1	-
Chest pain	0	-
Tachycardia	0	-
Abnormal ECG	0	-
Hypoxaemia	0	-
Myocardial infarct	0	-
Stroke	0	-

VTE, Venous thromboembolism; DVT, deep vein thrombosis; PE, pulmonary embolism; IQR, interquartile range; LMWH, Low molecular weight heparin; CTPA, Computed tomography pulmonary angiogram.

† , Median = 1; IQR, range = 1–1, 1–1.

Venous thromboembolism was confirmed in 10 patients out of the 11 patients who were treated for VTE. There was 1 incidental VTE diagnosed at staging CT scan.

## Discussion

Venous thromboembolism is a disease involving a variety of contributory factors. There are two main clinical subtypes that are associated with morbidity and mortality, DVT and PE.^1^

Venous thromboembolism carries a substantial burden and has been observed as a common complication in patients with active cancer. Its overall yearly rate is estimated to be roughly 6% – 8% although it differs across cancer types.^2^ In our study, the prevalence of VTE was 6.86%. Despite the difference in demographics and presence of medical conditions in the studies conducted before, the prevalence remains within range in our study. Venous thromboembolism is associated with major morbidity and mortality as well as a reduction in the well-being of patients.

Studies identified human immunodeficiency virus (HIV) and tuberculosis (TB) as risk factors for the development of VTE. In our study, 40% of those with VTE had HIV and they were all virally suppressed. TB was not observed in our study. In a study by P Moodley, et al.,^7^ the prevalence of HIV and TB were 53.0% and 21.2%, respectively.

Some studies^8,9^ have reported that smoking is a risk factor for VTE while in all our patients who had VTE, they were all non-smokers. Perhaps this is because our study was not population-based but of a small cohort of patients with a diagnosis of female gynaecological malignancies.

Moreover, 40% of patients who had VTE were above 51 years while 60% were already in menopause. Menopause had proven to be a contributory risk factor with a crude odd ratio of 0.81. A Korean study conducted by Yuk JS reported that older patients are at risk of DVT more than younger ones.^9^ Most of the patients (80%) with VTE had cervical cancer which is one of the common gynaecological cancer types in our setting. The cancer staging did not show a significant impact on the risk for VTE but fewer patients (10%) had cancer stage 2 while the other stages were all 30%.^9^ Matsou et al.^10^ reported a cumulative incidence of VTE in cervical cancer of 11.3% but in our study sample it was 44.8%. However, their study design and population was not like ours.

According to this study, 71% of the patients with VTE were postoperative which is a significant contributor to the development of VTE. Chemotherapy exposure did not increase the risk of VTE development in our study; only one patient was post-chemotherapy. Of the medical disorders, only 2 patients out of 10 were hypertensive and all the patients did not have any other comorbidities such as diabetes mellitus, hypercholesterolemia and cardiac diseases which are a risk in some of the RAMs.

In this study, most patients presented with unilateral leg swelling (70%) which is higher than in the study conducted by Snyman in South Africa. The clinical presentation was both pain and swelling of the limb in 60 participants (60.1%). Venous thromboembolism was mainly diagnosed with Doppler ultrasound (54.5%).^11^ One patient presented with shortness of breath and CTPA was performed, and it positively diagnosed VTE. Computed tomography scan was performed in 36.4% of the patients with positive VTE diagnosed. All patients for whom PE was suspected were put on therapeutic dosage of LMWH (1 mg/kg BD) which is the correct use of anticoagulants. In a South African study by Snyman, there was incorrect use of thromboprophylaxis and inadequate use of RAMs. In our population group, the RAMs were not compared before and in this study; it showed the Caprini score performing better than the other RAMs. This is mainly because in our population, less patients have cardiopulmonary diseases. Also, lifestyle habits such as smoking are mainly for men and a small fraction of women, as proven in our study that none of the patients with malignancy were smokers.

To our knowledge, this study is unique in our setting in terms of comparing the performance of the three RAMs with regard to risk scoring for VTE and the need for thromboprophylaxis in gynaecologic oncology patients.

These RAMs were all tested on patients who are high risk, and sensitivity, specificity, PPV and NPV were tested on patients who were diagnosed radiologically for any form of VTE. This biased selection was opted to enable an almost equal comparison. The study by Wang et al., even though it was specific to hepatocellular cancer patients, showed that the sensitivity of Khorana is low.^12^ A systematic review and meta-analysis reported the Khorana score to be superior for prediction of VTE in overall cancer patients.^2^

However, the Khorona score is known to perform well on patients who had received chemotherapy where the variable for treatment improves the sensitivity. In our cohort, there were no patients who had received chemotherapy as these would have been referred to the medical oncology unit for takeover. Therefore, it best compares the original Khorana with the other two RAMs.

The 80% sensitivity of the Caprini (the best-performing RAM of the three) means that in a clinical setting, there will only be 20% of those patients who have VTE that would be missed. Venous thromboembolism is a lethal condition in general, and in patients with malignancy and other comorbid diseases, it is even more lethal. The perfect test would be the one with 100% or closest to 100% sensitivity. The sensitivity and positive and NPVs of the Caprini RAM are known to be higher than that of the Padua RAM (all *p* < 0.05) although the specificity was lower than that of the Padua RAM (*p* = 0.001).^5^ In most studies conducted outside of African populations, Caprini RAM does not have a good sensitivity. In hospitalised patients, the Caprini RAM may improve the efficacy of prophylaxis and as a result, reduce the risk of VTE and death associated with it.

Although the study was not designed to report on patients of African race and ethnicity, it is worth reporting that most patients seen during the study period with gynaecological malignancies were African/black and those with VTE. Therefore, although the different RAMs were not compared among the different races, it can be reported that Caprini was most sensitive in this predominantly African race. A South African study conducted in Cape Town reported that the majority of patient sample (97.1%) were determined to be at moderate or higher risk of VTE following objective risk assessment with the Caprini RAM.^13^

## Conclusion

We have identified that there is no perfect tool which is the most predictive (sensitive and specific with good NPV) risk assessment tool in our population that correctly assesses risk and informs the plan for prophylactic thrombolytics. However, the Caprini score performed better and proved to be the most ideal scoring system to be used in this clinical setting. It has the potential to reduce mortality associated with VTE in gynaecological cancer patients. Currently, the best-known risk stratification tool tested outside the African continent is the Khorana score, which assigns points to five clinical and pre-chemotherapy laboratory parameters. The Caprini score needs to be tested in the same population on a prospective and perhaps multicentre South African study.

## References

[1] Riondino S, Ferroni P, Zanzotto FM, Roselli M, Guadagni F. Predicting VTE in cancer patients: Candidate biomarkers and risk assessment models. Cancers (Basel). 2019;11(1):95. 10.3390/cancers11010095 30650562 PMC6356247

[2] Mulder FI, Candeloro M, Kamphuisen PW, et al. The Khorana score for prediction of venous thromboembolism in cancer patients: A systematic review and meta-analysis. Haematologica. 2019;104(6):1277–1287. 10.3324/haematol.2018.209114 30606788 PMC6545838

[3] Kawaguchi H, Kanemura M, Nakamura M, et al. High incidence of VTE in gynaecological cancer patients before surgery. Bull Osaka Med Coll. 2013;59(1):9–16.

[4] Nawapas P, Putsarat I, Suvanna A. Risk factor of deep vein thrombosis development among gynecologic cancer patients. Asian Pac J Cancer Care. 2018;3(1):5–11. 10.31557/apjcc.2018.3.1.5

[5] Liu X, Liu C, Chen X, Wu W, Lu G. Comparison between Caprini and Padua risk assessment models for hospitalized medical patients at risk for venous thromboembolism: A retrospective study. Interact CardioVasc Thorac Surg. 2016;23(4):538–543. 10.1093/icvts/ivw158 27297558

[6] Zhou H, Hu Y, Li X, et al.. Assessment of the risk of venous thromboembolism in medical inpatients using the Padua prediction score and Caprini risk assessment model. J Atheroscler Thromb. 2018;25(11):1091–1104. 10.5551/jat.43653 29540637 PMC6224205

[7] Moodley P, Martisan NA, Yoyimbana W, et al. Venous thromboembolic disease in adults admitted to hospital in a setting with a high burden of HIV and TB [homepage on the Internet]. 2022 [cited 2024 Nov]. Available from: https://hdl.handle.net/10520/ejc-m_ajtccm_v27_n3_a6 10.7196/AJTCCM.2021.v27i3.155 PMC8573812 34761207

[8] Cheng YJ, Liu ZH, Yao FJ, et al. Current and former smoking and risk for venous thromboembolism: A systematic review and meta-analysis. PLoS Med. 2013;10(9):e1001515. https://doi.org/10.1371%2Fjournal.pmed.1001515 24068896 10.1371/journal.pmed.1001515 PMC3775725

[9] Paulsen B, Gran VO, Severinsen MT, et al. Association of smoking and cancer with the risk of venous thromboembolism: The Scandinavian Thrombosis and Cancer cohort. Sci Rep. 2021;11:18752. 10.1038/s41598-021-98062-0 34548519 PMC8455552

[10] Matsuo K, Ross MS, Im DD, et al. Significance of venous thromboembolism in women with uterine carcinosarcoma. Gyneacol Oncol. 2018;148(2):267–274. 10.1016/j.ygyno.2017.11.036 PMC7529359 29248197

[11] Snyman LC, Potgieter J. Venous thromboembolism: Risk profile and management of prophylaxis in gynaecological surgery patients. S Afr J OG. 2014;20(3):76–79. 10.7196/sajog.490

[12] Wang Y, Attar BM, Fuentes HE, Yu J, Zhang H, Tafur AJ. Performance of Khorana risk score for prediction of venous thromboembolism in patients with hepatocellular carcinoma. Clin Appl Thrombo Hemostasis. 2018;24(3):471–476. 10.1177/1076029617699088 PMC6714645 28288526

[13] Wehmeyer A, Coetzee R, Mccartney J. Venous thromboembolism risk assessment and prophylaxis in hospitalised medical patients in the Cape Town metropole, South Africa. S Afr Med J. 2022;112(2):117–123. 10.7196/SAMJ.2022.v112i2.16040 35139994

